# Evaluation of Zirconia Surfaces after Strong-Acid Etching and Its Effects on the Shear Bond Strength of Dental Resin Cement

**DOI:** 10.1155/2019/3564275

**Published:** 2019-07-01

**Authors:** Yongsang Lee, Kyung Chul Oh, Na-Hong Kim, Hong-Seok Moon

**Affiliations:** ^1^Graduate Student, Department of Prosthodontics, College of Dentistry, Yonsei University, Seoul, Republic of Korea; ^2^Faculty, Department of Prosthodontics, Veterans Health Service Medical Center, Seoul, Republic of Korea; ^3^Clinical Research Assistant Professor, Department of Prosthodontics, College of Dentistry, Yonsei University, Seoul, Republic of Korea; ^4^Professor, Department of Prosthodontics, College of Dentistry, Yonsei University, Seoul, Republic of Korea

## Abstract

The present study was intended to investigate changes in the microstructure and phase transformation of zirconia surfaces using etching and airborne-particle abrasion (AB) and the effects of these processes on the shear bond strength of dental resin cements to zirconia. Four groups were classified according to the surface treatment as follows: etching for 15 min (ET15), etching for 30 min (ET30), AB, and etching for 15 min following AB (ABET). These groups were then classified into two subgroups (a total of 8 groups with 11 specimens/group) according to the resin cement used for bonding, namely, Rely-X U200 (3M ESPE, St. Paul, MN, USA) or Panavia F 2.0 (Kuraray, Kurashiki, Okayama, Japan). Shear bond strength testing was performed using a universal testing device. Scanning electron microscopy (SEM) and X-ray diffraction (XRD) were also performed. When using Rely-X U200, ET15 exhibited the highest mean shear bond strength; the strengths of ET15, ABET, and ET30 were significantly higher than that of AB. When using Panavia F 2.0, ABET demonstrated the highest mean shear bond strength; the strengths of ABET and ET15 were significantly higher than those of ET30 and AB. The etched surface of zirconia could be observed using SEM, and the phase transformations resulting from each surface treatment were detected by XRD. Strong-acid etching of zirconia induced significant surface changes that increased the shear bond strength of resin cement, and the resin adhesive strength was higher when zirconia was etched with strong acid vs. AB alone.

## 1. Introduction

The production of dental prostheses using zirconia has been increasing in recent years. Zirconia exhibits mechanical properties comparable with those of metal dental materials, has a color similar to that of teeth, and has several physical and biocompatible advantages [1].

Many studies investigating adhesion with zirconia have been performed, and some adhesion enhancement has been confirmed using several different surface treatments. Examples of such surface treatments for zirconia include airborne-particle abrasion (AB) [2–4], silica coating [5, 6], selective infiltration etching [7], and laser etching [5, 6], among others. However, other studies have reported limitations in such methods. For instance, silica coatings are reportedly insufficient for long-term stability due to the hydrolytic degradation of silica coatings [8, 9]. Selective infiltration etching has a couple of clinical problems, including its complexity and the high costs that are associated with the application process. Laser etching is also reportedly less efficient at altering the surface of zirconia than is AB, exhibits lower adhesive strength when dental resin cements are applied, and causes phase transformation into the excessive monoclinic phase [10, 11].

It is expected that if AB and 10-methacryloyloxydecyl dihydrogen phosphate- (10-MDP-) containing luting agents are used adequately for cementing zirconia, then this will yield successful long-term clinical bonding [12–16]. However, it has also been suggested that the surface roughness of zirconia varies according to the particle size, distance, and duration of AB, which are manual processes and may affect the bonding strength of the resin adhesive [17]. In addition, a few studies have reported a decrease in the physical strength of zirconia depending on the flaws caused by AB [18–20].

Recently, studies examining the efficacy of employing chemical etching as a surface treatment for zirconia have been published [21, 22]. Especially, hydrofluoric acid has been reported to be useful for surface treatment of zirconia and resin cement bonding [23–25]. However, extensive investigations of this approach are lacking still. Accordingly, the aim of the present study was to investigate changes in the microstructure and phase transformation of zirconia surfaces using etching and AB, and in each case, it was determined whether it affected the shear bond strength of dental resin cements to zirconia. The null hypothesis was that a strong-acid solution would not be able to appropriately etch the zirconia surface for improving the shear bond strength of dental resin cements to zirconia.

## 2. Materials and Methods

Four groups were classified according to the surface treatment as follows: etching for 15 min (ET15), etching for 30 min (ET30), AB, and etching for 15 min following AB (ABET). These four groups were then classified into two subgroups each according to the resin cement used for bonding, i.e., Rely-X U200 (3M ESPE, St. Paul, MN, USA) or Panavia F 2.0 (Kuraray, Kurashiki, Okayama, Japan). A total of 8 groups were designated according to the zirconia surface treatment method and the resin cement used for bonding zirconia and the composite resin block. A total of 88 specimens were fabricated (Table 1), with 11 specimens per group for the shear bond strength testing.

### 2.1. Zirconia Block

**Figure 1:**
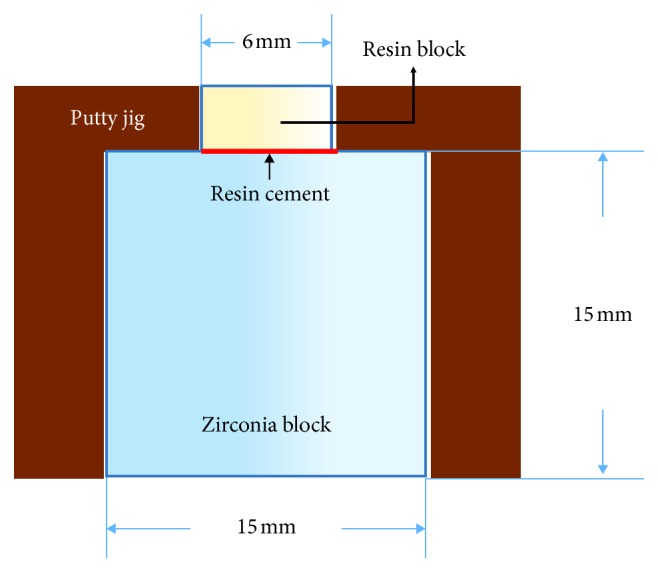
Cementing diagram of the zirconia block and composite resin block.

The Zircose E block (M&C Dental Co., Eunjin Chemical Co., Seoul, Korea) used in this study is a zirconium dioxide partially stabilized with 3 mol% yttria (Table 2). The sintering of the zirconia block was performed by a programmed furnace (Ceramill therm 3, Amann Girrbach, Koblach, Austria) and reached at the 1550°C of highest temperature and cooled to a temperature below 100°C in the furnace for reducing residual stress. The fully sintered zirconia block was cylindrical in shape, with a diameter of 15 mm and a height of 15 mm (Figure 1).

### 2.2. Resin Block

To fabricate the composite resin block that would be bonded to the surface of the zirconia, Filteck Z350 (3M ESPE) was poured into a cylindrical (6 mm inner diameter), polypropylene tube (SEOIL Industrial Co., Zanesville, OH, USA) and photopolymerized with a light-curing gun (S Lite, Shinwon Dental, Seoul, Korea) of 1,000 mW/cm^2^ intensity for 20 s. After removing the cylindrical resin block from the tube, the resin block was photopolymerized for an additional 20 s and adjusted to a cylinder height of 3 mm (Figure 1).

### 2.3. Airborne-Particle Abrasion

Using an AB device (Basic master; Renfert GmbH, Hilzingen, Germany) and a 110 *μ*m Al_2_O_3_ (Cobra aluminum oxide; Renfert GmbH) particle, zirconia surfaces were abraded at a pressure of 2 bar at a distance of 10 mm for 10 s. After AB, the zirconia blocks were immersed in 96% isopropyl alcohol, sonically cleaned for 3 min, and thoroughly washed again with running distilled water.

### 2.4. Etching

The zirconia block was etched using a strongly acidic solution, which was prepared by mixing 70% nitric acid (HNO_3_) and 48% hydrofluoric acid and adding hydrogen peroxide (H_2_O_2_) to achieve a 10 wt.% mixed solution. The zirconia block was immersed in the etching solution and etched for 15 min or 30 min while being sonicated at a frequency of 30 kHz and a power of 100 W/cm^2^ at room temperature. After the zirconia block was thoroughly washed with running distilled water, annealing was performed in the furnace heated to 1150°C for 1 h to completely remove the etchant and to reduce the residual stress that was incurred during the sintering process.

It has recently been reported that strong acid can be used to alter the surface of zirconia [21, 23–26]. In this study, the fabrication and application of strong acids were devised based on the results of previous studies investigating the etching of zirconia.

### 2.5. Cementation

To apply resin cement between the surface-treated zirconia block and composite resin block, Z-prime plus (BISCO, Inc., Schaumburg, IL, USA) was first applied to the zirconia block surface and then air was gently applied using a three-way dental syringe. Following this, Rely-X U200 resin cement was applied according to the manufacturer's instructions. Alternatively, after applying Clearfil ceramic primer (Kuraray) in the same manner, adhesion was performed using Panavia F 2.0 according to the manufacturer's instructions. The cementation jig was made from putty (3M ESPE), and resin blocks were bonded to the center of the zirconia block using the cementation jig (Figure 1). While the resin cement autopolymerized, a weight of 1 kg was applied as static loading to the putty jig for 5 min. Subsequently, the specimen from the putty jig was separated and excess resin cement around the resin block was removed carefully using a technical dental scalpel, and photopolymerization was performed for an additional 20 s.

### 2.6. Artificial Aging

A thermocycler (KD-TCS30; Kwang-duk FA, Gwangju, Korea) was used to artificially age the cemented zirconia-composite resin specimens for 5000 cycles between 5°C and 55°C. The mooring time at each temperature was 15 s, and the wait time was 2 s.

### 2.7. Shear Bond Strength

Immediately after the artificial aging process, the shear bond strength was measured using a universal testing device (Instron 3366; Instron Corporation, Seoul, Korea) with a crosshead speed of 0.5 mm/min at the site of approximately 1 mm away from the zirconia surface until the adhered composite resin block fell off.

### 2.8. Scanning Electron Microscopy

The zirconia surfaces were assessed using a scanning electron microscope (Hitachi S-3000N; Hitachi Co., Tokyo, Japan) at a magnification of 2,000x before and after the four surface treatments. The adhesion failure mode of the zirconia surface was assessed at a magnification of 40x.

### 2.9. X-Ray Diffraction

X-ray diffraction (XRD) experiments were performed to investigate the phase transformation of zirconia surface particles following the surface treatments. For this purpose, eight zirconia disks (1.5 mm thick, 15 mm in diameter) were prepared. One untreated disk and seven disks from each of the surface treatments were observed and analyzed.

### 2.10. Statistical Analysis

For the comparison of the shear bond strengths, one-way analyses of variance with the Dunnett T3 validation method were performed using PASW version 18.0 (IBM Corporation/SPSS Inc., Armonk, NY, USA) for Windows (Microsoft Corporation, Redmond, WA, USA). Differences were considered statistically significant at *P* ≤ 0.05.

## 3. Results

### 3.1. Shear Bond Strength

The mean and standard deviation of the shear bond strength between the dental resin cement and zirconia according to the surface treatment method are summarized in Table 3.

According to the results of our analyses of variance, the shear bond strengths were different according to the zirconia surface treatment for both cements (Table 3). When Rely-X U200 was used, the shear bond strength of ET15 exhibited the highest mean shear bond strength, and the mean shear bond strengths of ET15, ABET, and ET30 were significantly higher than that of AB (*P*=0.000, *F* = 17.15). When Panavia F 2.0 was used, the mean shear bond strength of ABET was the highest, and the mean shear bond strengths of ABET and ET15 were significantly higher than those of ET30 and AB (*P*=0.000, *F* = 21.51).

### 3.2. Scanning Electron Microscopy

**Figure 2:**
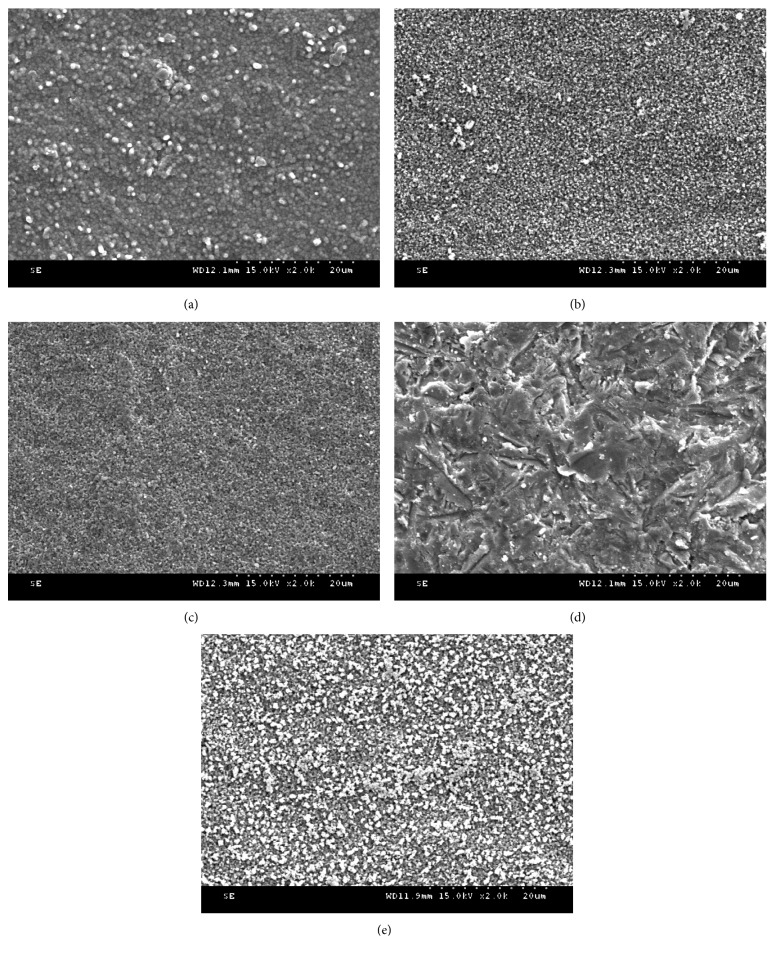
Scanning electron microscopy images after each surface treatment: (a) nontreatment; (b) etching for 15 min; (c) etching for 30 min; (d) treatment with 110 *μ*m Al_2_O_3_ airborne-particle abrasion; (e) treatment with 110 *μ*m Al_2_O_3_ airborne-particle abrasion and etching for 15 min (magnification ×2,000).

Our SEM evaluations revealed that the surface roughness of the zirconia was greater when the surface was treated with etching and AB than it was when zirconia did not undergo surface treatment. The appearances of the etched and airborne-particle-abraded surfaces were different from each other. And the irregularity of the etched surface was more uniform than that of the AB surface at 2,000x magnification (Figure 2).

### 3.3. Failure Mode

**Figure 3:**
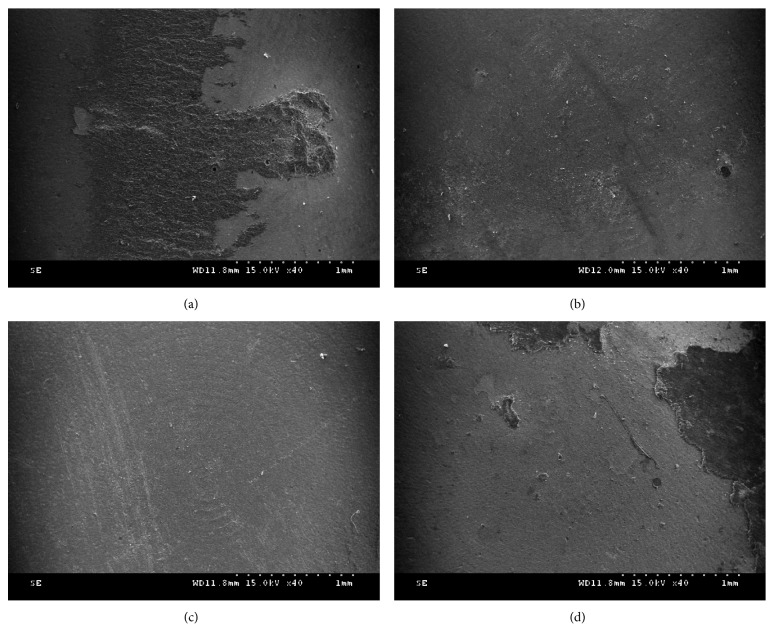
Scanning electron microscopy images after the shear bond strength test with Instron: (a) etching for 15 min (ET15-U; Rely-X U200 (3M ESPE, St. Paul, MN, USA)); (b) etching for 30 min (ET30-F; Panavia F 2.0 (Kuraray, Okayama, Japan)); (c) airborne-particle abrasion (AB-F; Panavia F 2.0); (d) airborne-particle abrasion, followed by etching for 15 min (ABET-U; Rely-X U200) (magnification ×40).

**Figure 4:**
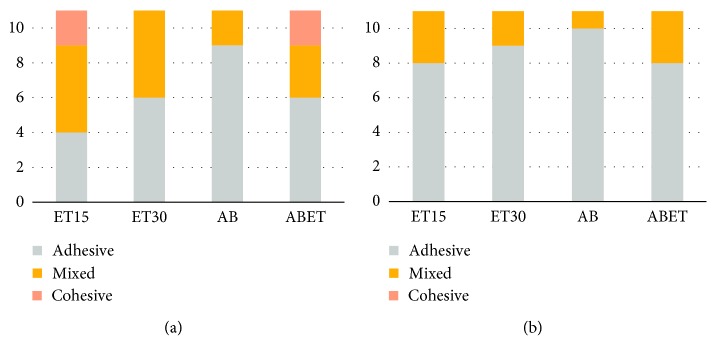
Failure modes of each group after shear bond tests: (a) when using Rely-X U200; (b) when using Panavia F 2.0.

After the shear bond strength testing of the dental resin cements to zirconia, the zirconia surface was observed at 40x magnification using SEM (Figure 3). In the ET15 and ABET when using Rely-X U200, mixed and cohesive failures were observed, while adhesive failure was primarily observed in the other groups (Figure 4).

### 3.4. X-Ray Diffraction

**Figure 5:**
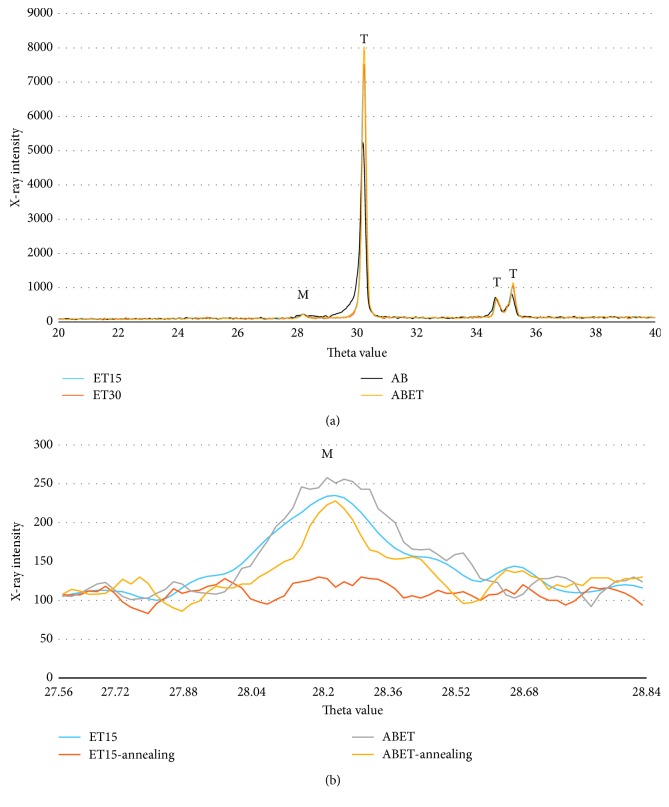
XRD patterns before and after annealing following surface treatment: (a) XRD pattern between 2*θ* values of 20 and 40; (b) main peak of the monoclinic phase on the XRD pattern between 2*θ* values of 27.56 and 28.88.

The ET15, ET30, AB, and ABET samples exhibited phase transformations of 2.8%, 3.6%, 3.5%, and 5%, respectively, into the monoclinic phase after surface treatment; the monoclinic phase of the ET15, ET30, and ABET samples was reduced to 0%, 0%, and 3.1%, respectively, after annealing. In the XRD pattern, the peak that appeared when the two-theta (*θ*) value was approximately 28° represents the main peak of the monoclinic phase (Figure 5).

## 4. Discussion

According to the results of this study (Table 3), the null hypothesis stating that zirconia surfaces cannot be appropriately etched using strong-acid solutions for improving the shear bond strength of dental resin cements to zirconia was rejected.

The etching process involves chemically dissolving particles on the zirconia surface by applying a strong acid, which may be advantageous because it permits a more-objective application and yields more-consistent results than AB. Similar to previous studies, our SEM images confirmed that morphological changes occurred on the surface of zirconia following etching with a strongly acidic solution. The surface irregularities of samples that were etched with acid were more uniform and detailed than those in the samples that were treated with AB alone (Figure 2). In addition, the surfaces of ET30 samples were over-etched compared with those of ET15 samples, and the surface roughness of ET30 samples was lower than that of ET15 samples, likely leading to the lower shear bond strength of the ET30 vs. ET15 group.

The shear bond strength of the AB group in the present study was rather low compared to that reported in other studies [3, 4, 8, 27]. This seemed to be due to the fact that the initial surface condition of the zirconia before surface treatment and the distance between the crosshead of Instron and zirconia surface was different from those of previous studies [28]. In previous experiments, zirconia in a semisintered state was cut into blocks of a specific shape using a diamond bur or milling machine, and the surfaces of the block for bonding were prepared using sandpaper processing; then, the block was fully sintered. However, in clinical practice, it is believed that sandpaper could not be applied to the inner surface of the prosthesis for bonding, and thus, we thought that using computer numerical control milling would be more suitable for preparing the surface, as this approach is similar to that used in clinical situations. However, methodological verification of this approach to surface preparation is necessary.

Results from the XRD experiments in the present study revealed that, in the ABET samples, 3.1% of the monoclinic phase of zirconia remained after annealing (Figure 5). It was speculated that the flexural strength of ABET zirconia may be enhanced by the transformation toughening [29, 30].

The average shear bond strength of samples on which Rely-X U200 was used was higher than that of samples on which Panavia F 2.0 was used in the ET15, ET30, and ABET groups, which is in agreement with previous studies [31, 32]. According to Oyagüe et al., the microtensile bond strength of the self-adhesive resin cement (Rely-X Unicem®) was found to be higher in all situations than that of conventional (Calibra®) and self-etching resin cements (Clearfil Esthetic Cement®). The authors speculated that this was because the self-adhesive cement penetrates more easily through gaps in the roughened surface to form microchemical interlocks and because the inorganic filler of the self-adhesive resin cement is more resistant to hydrolysis and plays an important role in cement formation. Magne et al. [8] reported that the methacrylate group contained in resin cement binds to the methacrylate of the primer, which concurs with the results of the present study in that the combination of Z-prime plus and Rely-X U200 was better than that of Clearfil ceramic primer and Panavia F 2.0.

There are some advantages of the etching of zirconia beyond more objectively and consistently increasing bond strength of cement. One-time procedure of etching zirconia with strong acid(s) can be used not only for bonding with resin cement but also for increasing the bonding strength with porcelain veneers at the same time, and there will be no need to perform airbone-particle abrasion in the clinic before cementation.

## 5. Conclusions

Within the limitations of the present study, the following conclusions were drawn: (1) strong-acid etching of zirconia caused significant surface changes that increased the shear bond strength of resin cement, and (2) the shear bond strength of resin cements was higher when zirconia was etched with strong acid than when AB was used alone.

## Figures and Tables

**Table 1:** Experimental group allocation for measuring the shear bond strength.

Resin cement (group)	Surface treatment			
	Etching for 15 min (ET15)	Etching for 30 min (ET30)	Airborne-particle abrasion (AB)	Airborne-particle abrasion and etching for 15 min (ABET)
Rely-X U200^*∗*^ (U)	ET15-U	ET30-U	AB-U	ABET-U
Panavia F 2.0^†^ (F)	ET15-F	ET30-F	AB-U	ABET-U

^*∗*^3M ESPE, St. Paul, MN, USA; ^†^Kuraray, Kurashiki, Okayama, Japan.

**Table 2:** Materials used in the present study and their characteristics.

Material	Manufacturer	Trade name		Main composition
Zirconia block	M&C Dental Co., Seoul, Korea	Zircose-E block		ZrO_2_ (89.86%), Y_2_O_3_ (5.7%), HfO_2_ (4.29%)
Zirconia primer	Bisco Dental, Schaumberg, IL, USA	Z-prime plus		Organophosphate monomer (MDP), carboxylic acid monomer (BPDM), HEMA, ethanol
Zirconia primer	Kuraray, Okayama, Japan	Clearfil ceramic primer		3-Methacryloxypropyl trimethoxy silane, MDP, ethanol
Composite resin	3M ESPE, St. Paul, MN, USA	Filteck Z350		Bis-PMA, DUDMA, Bis-GMA, TEGDMA, ZrO_2_/SiO_2_ nanocluster, SiO_2_ nanofiller
Resin cement	3M ESPE, St. Paul, MN, USA	Rely-X U200		Base: fiberglass, ester, phosphoric acid, methacrylate, TEGDMA, silanated silica and persulfate, and inorganic fillers (45% wt) Catalyst: fiberglass, substitute dimethacrylate, silanated silica, sodium *p*-toluenesulfonate, and calcium hydroxide
Resin cement	Kuraray, Okayama, Japan	Panavia F 2.0	Paste A	BPEDMA, MDP, DMA, silica, barium, sulfate, dibenzoylperoxide
			Paste B	*N*,*N*-Diethanol-*p*-toluidine, silica sodium fluoride

MDP: 10-methacryloyloxydecyl dihydrogen phosphate; BPDM: biphenyl dimethacrylate; HEMA: hydroxyethyl methacrylate; Bis-PMA: propoxylated bisphenol A-dimethacrylate; DUDMA: diurethane dimethacrylate; Bis-GMA: bisphenol A-glycidyl methacrylate; TEGDMA: triethylene glycol dimethacrylate; BPEDMA: bisphenol A-polyethoxy dimethacrylate; DMA: bisphenol A-polyethoxy dimethacrylate.

**Table 3:** Mean, standard deviation (SD), standard error (SE), and 95% confidence interval (CI) of each of the four groups according to surface treatment when using Rely-X U200^*∗*^ resin cement (MPa) or using Panavia F 2.0^†^ resin cement (MPa).

	Group	*n*	Mean	SD	SE	95% CI	
						Min.	Max.
Rely-X U200^*∗*^	ET15	11	13.8^a^	2.8	0.9	11.9	15.7
	ET30	11	12.2^a^	4.9	1.5	8.9	15.6
	Ab	11	3.9^b^	2.1	0.6	2.5	5.3
	ABET	11	13.3^a^	4.3	1.3	10.4	16.9
	Total	44	10.8	5.4	0.8	9.1	12.4

Panavia F 2.0^†^	ET15	11	9.7^i^	2.5	0.8	8	11.4
	ET30	11	6.1^ii^	1.2	0.4	5.2	6.9
	AB	11	4.5^ii^	1.5	0.5	3.5	5.5
	ABET	11	11.2^i^	3.1	0.9	9.1	13.2
	Total	44	7.9	3.5	0.5	6.8	8.9

^*∗*^3M ESPE, St. Paul, MN, USA; ^†^Kuraray, Kurashiki, Okayama, Japan; ET15, etched for 15 min; ET30, etched for 30 min; AB, airborne-particle abrasion; ABET, airborne-particle abrasion followed by etching for 15 min. Superscript letters “a” and “b” indicate statistically significant differences from each other and also for i and ii (*P* ≤ 0.05).

## Data Availability

The data are available at https://drive.google.com/drive/folders/1CRCsiFoseJ17k4-ItbkCIDbr2Rvgd2tZ.
